# Angiopoietin-2 Is an Early Indicator of Acute Pancreatic-Renal Syndrome in Patients with Acute Pancreatitis

**DOI:** 10.1155/2016/5780903

**Published:** 2016-02-28

**Authors:** Mateusz Sporek, Paulina Dumnicka, Agnieszka Gala-Bladzinska, Piotr Ceranowicz, Zygmunt Warzecha, Artur Dembinski, Ewa Stepien, Jerzy Walocha, Ryszard Drozdz, Marek Kuzniewski, Beata Kusnierz-Cabala

**Affiliations:** ^1^Surgery Department, The District Hospital, 22 Szpitalna Street, 34-200 Sucha Beskidzka, Poland; ^2^Department of Anatomy, Faculty of Medicine, Jagiellonian University Medical College, 12 Kopernika Street, 31-034 Krakow, Poland; ^3^Department of Medical Diagnostics, Faculty of Pharmacy, Jagiellonian University Medical College, 9 Medyczna Street, 30-688 Krakow, Poland; ^4^St'Queen Jadwiga Clinical District Hospital No. 2, 60 Lwowska Street, 35-301 Rzeszow, Poland; ^5^Department of Physiology, Faculty of Medicine, Jagiellonian University Medical College, 16 Grzegorzecka Street, 31-531 Krakow, Poland; ^6^Institute of Physics, Department of Medical Physics, Faculty of Physics, Astronomy and Applied Computer Science, Jagiellonian University, 11 Lojasiewicza Street, 30-348 Krakow, Poland; ^7^Department of Nephrology, Faculty of Medicine, Jagiellonian University Medical College, 15c Kopernika Street, 31-501 Krakow, Poland; ^8^Department of Diagnostics, Chair of Clinical Biochemistry, Faculty of Medicine, Jagiellonian University Medical College, 15a Kopernika Street, 31-501 Krakow, Poland

## Abstract

Within the first week of the disease, acute kidney injury (AKI) is among the most common causes of mortality in acute pancreatitis (AP). Recently, serum angiopoietin-2 (Ang-2) has been associated with hyperdynamic state of the systemic circulation. The aim of this study was to examine the associations between Ang-2 and the clinical AP severity during the first 72 hours of the disease, and organ disfunction, including AKI.* Methods*. Study included patients admitted to the surgery ward, diagnosed with AP. AKI was diagnosed according to KDIGO guidelines and renal failure according to modified Marshall scoring system. Ang-2 was determined in serum with ELISA.* Results*. AP was classified as mild (MAP) in 71% of patients, moderately severe (MSAP) in 22%, and severe (SAP) in 8%. During the first 72 hours of AP, 11 patients developed AKI and 6 developed renal failure. Ang-2 at 24, 48, and 72 hours following the onset of AP symptoms significantly predicted SAP and MSAP, as well as AKI and renal failure. Also, Ang-2 significantly correlated with acute phase proteins as well as with the indicators of renal disfunction.* Conclusions*. Serum Ang-2 may be a relevant predictor of AP severity, in particular of the development of AP-renal syndrome.

## 1. Introduction

Destabilization of the vascular endothelium as well as escape of fluid from the vascular bed is one of the main factors leading to vasodilatation, decrease in blood pressure, and the development of early complications of acute pancreatitis (AP), which clinically manifest themselves as shock and developing organ failure [1, 2]. The patient's transfer to the intensive care unit, initiation of intensive fluid resuscitation within the first 12–24 hours of the onset of severe acute pancreatitis (SAP), close monitoring of organ function, and, in case of acute kidney injury (AKI) in circulatory unstable patients, implementation of continuous blood purification techniques (continuous venovenous hemofiltration or continuous venovenous hemodiafiltration) may have a decisive impact on the survival in this group of patients [1, 3, 4].

Such procedures are particularly justified because, in nearly half of patients classified as SAP, organ failure is diagnosed on admission, whereas the other 50% of patients develop symptoms of organ failure in the first four days of hospitalization [5, 6]. Moreover, more than a half of deaths in SAP patients occur within the first week of the disease [7]. AKI and acute respiratory distress syndrome are the most common causes of mortality (70–80%) in this group of patients [4, 8].

Currently, for the diagnostics of AKI, the risk, injury, failure, loss, and end stage (RIFLE) and the Kidney Disease Improving Global Outcomes (KDIGO) criteria are used. Both criteria are based on the increase in serum creatinine and the decrease in diuresis [4, 9]. In the treatment of AKI, it is critical to quickly determine the cause of kidney injury (prerenal, intrinsic, or postrenal). Unfortunately, an increase in serum creatinine concentration usually occurs about 1-2 days after kidney injury, which often delays the possibility of taking action in the reversible phase of the disease. The limited value of serum creatinine in the early phase of AKI as well as a burdensome necessity to monitor diuresis requiring (invasive) urinary catheterization urges us to search for new biomarkers of kidney injury. Early indicators include urine concentrations of neutrophil gelatinase-associated lipocalin (uNGAL), kidney injury molecule-1 (KIM-1), and interleukin-18 (IL-18) [9].

One of the promising prognostic biomarkers of acute pancreatitis is angiopoietin-2 (Ang-2) [10]. Studies by Whitcomb et al. [11] showed that Ang-2 concentration is considerably increased already at the time of the diagnosis of AP and is significantly higher in patients with developing persistent organ failure [11]. Watanabe et al. [12] demonstrated a correlation between Ang-2 concentrations and perfusion parameters using computed tomography [12]. Increased Ang-2 concentrations in blood are associated with hyperdynamic state of the systemic circulation, and Ang-2 monitoring may be helpful in the evaluation of the response to systemic treatment, that is, fluid resuscitation, or antithrombotic therapy [12].

Ang-2 belongs to a new class of angiogenic growth factors that exerts a selective effect on the endothelium by binding with Tie-2 receptor. It was demonstrated that Ang-2 has an inhibitory effect on angiopoietin-1 binding to Tie-2, which leads to destabilization of the vascular endothelium, increased fluid leakage, and leukocyte adhesion [2, 12, 13]. Under physiologic conditions, Ang-2 is stored within Weibel-Palade bodies, along with P-selectin, von Willebrand factor, CD63, interleukin-8, and endothelin-1 [14]. Factors such as hypoxia, inflammation, or mechanical injury may cause the rapid release of Ang-2 into the systemic circulation [13, 14]. Ang-2 was proposed to be a link between angiogenic and inflammatory pathway [14]. Serum concentrations of Ang-2 seem to reflect the extent of endothelial activation [2] and may be useful in early clinical assessment of patients with acute conditions, including AP.

The aim of this preliminary study was to assess Ang-2 serum concentrations in patients with AP of various severity in the early phase of the disease, that is, during the first 72 hours of the disease, as well as to examine the associations between Ang-2 and the clinical measures of the severity of AP, in particular organ dysfunction, including AKI.

## 2. Materials and Methods

The prospective observational study included 65 consecutive adult patients (men and women) admitted with AP, hospitalized, and treated in the Department of Surgery, District Hospital in Sucha Beskidzka, Poland, between January and December 2014. AP was diagnosed according to the 2012 revision of the Atlanta classification, that is, when at least two of the three following features were present: abdominal pain consistent with AP (acute onset and persistent and severe and epigastric pain); serum lipase or amylase activity at least three times greater than the upper reference limit; and the characteristic findings of AP on contrast-enhanced computer tomography or magnetic resonance imaging or transabdominal ultrasonography [15]. Only adult patients who signed the informed consent for the study were included. Patients with symptoms of AP lasting longer than 24 hours were excluded. Also, the exclusion criteria were chronic pancreatitis, neoplasms, and chronic liver diseases such as cirrhosis or viral hepatitis.

On the basis of the clinical evaluation of the severity of AP, the patients were assigned to one of the 3 groups: with mild acute pancreatitis (MAP), moderately severe acute pancreatitis (MSAP), or severe acute pancreatitis (SAP). The MAP group included patients who did not show any organ failure or local complications. Patients with organ failure lasting less than 48 hours (transient organ failure), local complications (necrosis, acute necrotic collection, and walled-off pancreatic necrosis), and/or exacerbation of comorbidity were assigned to MSAP. Patients with persistent organ failure (lasting more than 48 hours) and ≥1 local complications were assigned to SAP [15].

AKI was diagnosed according to KDIGO guideline [16], that is, when serum creatinine increased ≥26.5 *μ*mol/L within 48 hours or ≥1.5 times which is known or presumed to occur within 7 days, or urine volume <0.5 mL/kg/h for 6 hours. Organ failure was diagnosed according to the modified Marshall scoring system (MMSS), as cited in 2012 revision of the Atlanta classification [15]; in particular, renal failure was diagnosed in patients with serum creatinine ≥170 *μ*mol/L.

In order to determine serum Ang-2 concentrations in healthy persons, a control group was recruited which included 21 healthy volunteers (men and women) at the age similar to that of the study group, without any pancreatic, liver, or renal diseases.

The study protocol was approved by the Bioethics Committee of the Jagiellonian University (Approval number KBET/247/B/2013).

### 2.1. Laboratory Tests

Blood and urine for laboratory tests were collected at 24, 48, and 72 hours from the onset of symptoms of AP. The routine laboratory tests and the measurements of uNGAL, urine albumin, and urine creatinine were performed at the day of collection, while serum samples for serum NGAL (sNGAL) and Ang-2 measurements were frozen in aliquots and stored in −70°C. The routine tests included complete blood count, urinalysis, serum amylase, total calcium, albumin, urea, creatinine, glucose, C-reactive protein, plasma fibrinogen, and D-dimer. All the routine laboratory tests and the measurements of uNGAL, urine albumin, and urine creatinine were performed in the Department of Laboratory Diagnostics, District Hospital in Sucha Beskidzka, Poland. Complete blood counts were analyzed in K_2_EDTA-anticoagulated blood with the Sysmex XE 2100 (Sysmex Corp., Japan) automated hematology analyzer. Urine NGAL concentrations were determined with the ARCHITECT Analyzer (Abbott Park, USA) using chemiluminescent microparticle immunoassay. Ang-2 and sNGAL measurements were performed in the Department of Diagnostics, Chair of Biochemistry, Jagiellonian University Medical College in Cracow, Poland. Serum NGAL concentrations were measured with the Human Lipocalin-2/NGAL ELISA kits (BioVendor, Brno, Czech Republic). Serum Ang-2 were measured with the Quantikine ELISA Human Angiopoietin-2 Immunoassay kits (R&D Systems, Minneapolis, USA).

### 2.2. Statistical Analysis

Data are shown as number of patients (percentage of the group) for categories and median (25th–75th percentile) or mean ± standard deviation for qualitative variables, depending on distribution (as assessed using the Shapiro-Wilk test). As the distributions of Ang-2 concentrations significantly differed from normal, the nonparametric tests were used. In detail, the differences between the groups were studied using the Kruskal-Wallis ANOVA or Mann-Whitney test, and the Spearman coefficient was used to assess correlations. In order to study associations between Ang-2 and the measures of AP severity, simple and multiple logistic regression were used, and the resulting odds ratios (ORs) were reported with 95% confidence intervals (95% CIs). Results were considered statistically significant at *p* < 0.05. The Statistica 10.0 (StatSoft, Tulsa, USA) software package was used for computations.

## 3. Results

In 71% of the study patients, AP was classified as mild, 22% had MSAP, and 8% had SAP (Table 1). In most patients, comorbidities were observed, most commonly hypertension (22 patients, 34%), ischemic heart disease (18 patients, 28%), and diabetes (10 patients, 15%); seven patients (11%) were diagnosed with lung diseases and 3 (5%) with kidney diseases (chronic kidney disease stages G3 in 2 patients and G4 in 4 patients). During the first 72 hours of AP, 11 patients developed AKI according to the KDIGO criteria, including stage 1 in 10 patients and stage 2 in one patient. According to the modified Marshall scoring system, renal failure was diagnosed in 6 patients. The detailed clinical characteristics of the patients are presented in Table 1.

**Figure 1:**
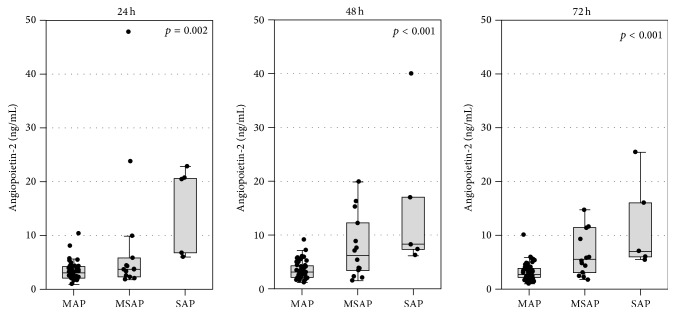
Serum angiopoietin-2 concentrations at 24, 48, and 72 hours after the onset of symptoms in patients with mild (MAP), moderately severe (MSAP), and severe acute pancreatitis (SAP). Data are shown as median, 25th–75th percentile (boxes), and nonoutlier range (whiskers); the points represent raw data.

The median serum concentrations of Ang-2 were about 2 times higher in the whole group of AP patients than in healthy controls (Table 2). However, the median Ang-2 concentrations in MSAP and especially in SAP patients were several times higher; the highest Ang-2 concentrations in those groups were observed in serum samples taken 24 hours after the onset of symptoms of AP (Figure 1). The median concentrations of urea, creatinine, and uNGAL in the whole group of patients were within the reference ranges (Table 2); however, 21 patients had an estimated glomerular filtration rate (eGFR, based on MDRD equation) of less than 60 mL/min/1.73 m^2^ at 24 hours. The concentrations of urea and creatinine were the highest at 24 hours and declined with treatment (Table 2).

**Figure 2:**
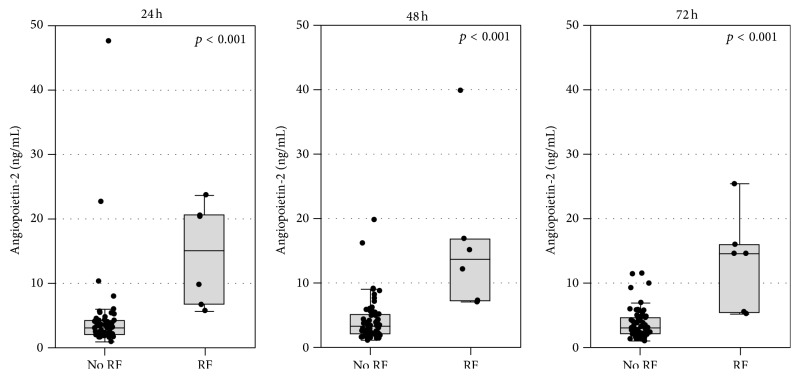
Serum angiopoietin-2 concentrations at 24, 48, and 72 hours after the onset of symptoms in AP patients without renal failure (no RF) and with renal failure (RF) diagnosed according to the modified Marshall scoring system. Data are shown as median, 25th–75th percentile (boxes), and nonoutlier range (whiskers); the points represent raw data.

Ang-2 concentrations in the first 72 hours of AP significantly predicted more severe disease (Table 3). In particular, Ang-2 was a significant predictor of AKI (diagnosed based on the KDIGO criteria) and renal failure (diagnosed according to the modified Marshall scoring system) both in simple analysis (Table 3) and after adjustment for age, sex, and comorbidities, with ORs for AKI of 1.13 (1.01–1.26); 1.40 (1.11–1.77); and 1.66 (1.18–12.32) and ORs for renal failure of 1.14 (1.02–1.28); 1.38 (1.10–1.74); and 1.84 (1.16–2.92) per 1 ng/mL increase in Ang-2 concentration at 24, 48, and 72 hours, respectively. The concentrations of Ang-2 in patients with a creatinine ≥170 *μ*mol/L (i.e., renal failure according to the modified Marshal scoring system) are shown in Figure 2.

During the first 72 hours of AP, serum Ang-2 concentrations were significantly correlated with several laboratory markers used in routine monitoring of AP patients, that is, hematocrit, CRP, fibrinogen, or D-dimer (Table 4). Also, Ang-2 negatively correlated with albumin and calcium concentrations at 48 and 72 hours (Table 4). Interestingly, Ang-2 concentrations were positively correlated with markers related to kidney function, that is, urea, sNGAL, and uNGAL, during the first 48 hours, as well as creatinine and urine albumin/creatinine ratio (uACR) during the entire study (Table 4).

## 4. Discussion

Acute pancreatitis is a relatively common disease that in most patients is mild or self-limiting; however, about 20% of patients develop SAP associated with life-threatening complications and a mortality of 20–30% [3, 9]. In older patients (over 60 years of age), being obese (with a body mass index greater than 30) or with chronic comorbidities such as cardiovascular diseases or chronic kidney disease, the mortality can reach 50–80% [4].

In the present study, serum Ang-2 concentrations significantly predicted the severity of AP in the early phase of the disease. Also, the correlations were found between Ang-2 and the laboratory markers of organ failure and inflammation. In particular, Ang-2 significantly correlated with the markers of kidney function and predicted both AKI diagnosed according to KDIGO definition and renal failure diagnosed according to modified Marshall scoring system. The most reliable indicator of the severity of AP is organ failure persisting more than 48 hours [17]. The 2012 revision of Atlanta classification recommends the assessment of organ failure in AP with the use of the modified Marshall scoring system [15]. Although the pathogenesis of AKI in SAP is not fully elucidated, AKI significantly contributes to mortality in AP. Especially susceptible are elderly patients with reduced glomerular filtration and patients with history of renal disease [16, 18]. In the revised Atlanta classification, a patient's age over 60 years and the presence of comorbidities are recognized factors that significantly worsen the prognosis and are associated with more severe AP; this should be taken into account in the preliminary assessment of patients [15].

The results of the present study are in concordance with the observations of Buddingh et al. [10] and Whitcomb et al. [11] who reported the association between high serum Ang-2 concentrations and SAP. Also, increased Ang-2 has been recently associated with AKI [19].

Ang-2 is found in endothelial cells at the sites of vascular remodeling, and its autocrine effect weakens the interaction between endothelial cells and the surrounding cells, especially pericytes [20]. Vascular endothelial growth factor (VEGF) participates in the regulation of Ang-2 synthesis [21]. In the initial phase of inflammation and in the absence of VEGF, Ang-2 causes vascular regression by inducing apoptosis of endothelial cells. In the systemic inflammatory process, activated neutrophils and monocytes release a range of enzymes such as phospholipase, elastase, or lipocalin-2, which intensifies degradation of phospholipids and may lead to pancreatic and peripancreatic necrosis, as well as intensified infiltration of other organs by inflammatory cells [4]. In the present study, the concentrations of Ang-2 in the systemic circulation were higher in patients than in controls and correlated with inflammatory markers, that is, CRP, fibrinogen, and NGAL concentrations in serum.

The renal endothelium is identified as a rich source of Ang-2. Its release from the kidneys may increase due to kidney injury, for example, in the course of AP [2, 14, 22, 23]. The correlations between Ang-2 and urea, creatinine, uNGAL, and albuminuria support the association of Ang-2 with kidney injury. Urine NGAL is a promising biomarker of AKI [24]. As a low molecular weight lipocalin, NGAL is easily filtered by healthy glomeruli and then almost completely absorbed in proximal tubules. The main fraction of uNGAL in AKI is synthesized in the distal tubules of the glomeruli in response to a damaging factor. In turn, increased sNGAL concentrations are observed as a consequence of either decreased glomerular filtration or increased synthesis by activated neutrophils during inflammation. In AP, various factors may lead to AKI, such as the effect of pancreatic amylase on the renal microcirculation, hypoxemia, or a toxic effect of excessively produced pancreatic phospholipase A_2_ on the proximal tubules. However, the primary cause of the development and progression of AKI in AP is associated with hypovolemia associated with a decrease in filtration pressure in the kidneys [4]. Hypovolemia is a result of a systemic inflammatory state initiated by AP, associated with vasodilation, escape of fluid into the third space, and increase in intra-abdominal pressure. In patients with SAP, intra-abdominal hypertension is observed, which significantly contributes to the reduced perfusion of organs, including the kidneys [25]. Dehydration and hypovolemia are also intensified by vomiting or fever, often accompanying AP. These factors cause a decrease in renal perfusion and, as a consequence, damage to the renal tubules and the increase in uNGAL concentrations.

In chronic kidney disease, albuminuria is a recognized indicator of renal function, assessed along with eGFR; uACR is recommended as a simple measure of albuminuria [18]. In AKI, albuminuria may be associated with an injury to the endothelium of glomerular vessels, being a part of a filtration barrier. The other mechanism responsible for albuminuria in AKI is an injury to the proximal tubule and reduced albumin reabsorption from primary urine. Recently, albuminuria observed during the first 72 hours of ICU stay has been associated with worse outcomes in patients with sepsis-associated AKI [26]. On the other hand, the meta-analyses performed by CKD prognosis consortium, published in 2015, have recognized albuminuria (uACR) as a risk factor for AKI [27, 28]. In the present study, uACR showed a positive correlation with serum Ang-2 concentrations during the first 48 hours following the onset of AP. Tsai et al. [14] and Chang et al. [29] reported the association between increased Ang-2 and albuminuria in patients with chronic kidney disease.

## 5. Conclusions

Early diagnosis and quick and accurate determination of the cause are critical for the effective treatment of AKI. The present study shows a correlation between increased serum Ang-2 concentrations in patients with AP and deteriorated renal function in the early phase of the disease. High Ang-2 concentrations correlated with uNGAL and uACR in patients with AP in the “therapeutic window,” that is, within the first 48 hours following the onset of AP. Thus, serum Ang-2 may be a relevant predictor of the development of acute pancreatic-renal syndrome and a useful tool for a clinician in the evaluation of disease severity.

## Figures and Tables

**Table 1:** Clinical characteristics of patients included in the study.

Variable	Patients with acute pancreatitis, *n* = 65
Male sex, *n* (%)	34 (52)
Age, years	62 ± 16
Etiology:	
Gallstones, *n* (%)	33 (51)
Alcohol, *n* (%)	18 (28)
Hypertriglyceridemia, *n* (%)	5 (8)
After ERCP, *n* (%)	1 (2)
Others, *n* (%)	8 (12)
Duration of pain until admission, hours	12 (6–24)^*∗*^
Duration of hospital stay, days	6 (5–12)^*∗*^
Severity:	
Mild acute pancreatitis, *n* (%)	46 (71)
Moderately severe acute pancreatitis, *n* (%)	14 (22)
Severe acute pancreatitis, *n* (%)	5 (8)
BISAP score:	
=2 in the first 24 hours, *n* (%)	12 (18)
≥3 in the first 24 hours, *n* (%)	6 (9)
Comorbidities, *n* (%)	50 (80)
SIRS, *n* (%)	8 (12)
Pancreatic or peripancreatic necrosis, *n* (%)	3 (5)
Peripancreatic fluid collections, *n* (%)	5 (8)
Transient organ failure, *n* (%)	6 (9)
Persistent organ failure, *n* (%)	5 (8)
Pleural effusion, *n* (%)	13 (20)
Acute kidney injury (KDIGO), *n* (%)	11 (17)
Renal failure (MMSS), *n* (%)	6 (9)
Antibiotic prophylaxis, *n* (%)	31 (48)
Parenteral nutrition, *n* (%)	3 (5)
Surgery, *n* (%)	3 (5)
Early mortality/late mortality, *n* (%)	0/3 (5)

^*∗*^Data are presented as median (lower–upper quartile).

*n* (%): number of patients in each category (percentage of the studied group of 65 patients); BISAP: bedside index for severity in acute pancreatitis; SIRS: systemic inflammatory response syndrome; ERCP: endoscopic retrograde cholangiopancreatography; MMSS: modified Marshall scoring system; KDIGO: Kidney Disease Improving Global Outcomes.

**Table 2:** Laboratory data in the whole group of patients with acute pancreatitis (*n* = 65).

Variable	24 hours	48 hours	72 hours	Reference values
Ang-2 [ng/mL]	3.18 (2.10–4.64)	3.33 (2.06–5.65)	3.12 (2.17–5.21)	1.73 ± 0.38 (range: 1.17–2.47)^*∗*^
CRP [mg/L]	14.00 (2.60–86.70)	119.70 (52.00–237.40)	104.80 (33.80–227.80)	<5.0
WBC [×10^3^/*µ*L]	11.21 (9.55–15.05)	9.80 (6.62–12.87)	8.43 (6.17–10.62)	4.00–10.00
HCT [%]	42.8 (39.1–45.5)	39.8 (35.1–42.8)	39.5 (35.4–42.2)	F: 37.0–47.0/M: 40.0–54.0
PLT [×10^3^/*µ*L]	234 (188–259)	197 (160–230)	206 (168–246)	150–350
Amylase [U/L]	1069 (618–1844)	162 (114–316)	92 (62–152)	62–220
Glucose [mmol/L]	7.81 (6.43–10.52)	5.39 (4.53–6.20)	5.28 (4.76–6.42)	3.3–5.6
Urea [mmol/L]	6.07 (4.31–7.97)	4.36 (3.35–7.33)	4.39 (3.35–5.44)	2.76–8.07
Creatinine [*µ*mol/L]	76.0 (65.7–99.1)	70.9 (62.4–90.7)	70.5 (57.7–89.1)	45.0–97.0
Fibrinogen [g/L]	2.80 (2.20–3.55)	3.87 (3.11–4.98)	4.49 (3.52–5.36)	1.8–3.5
D-dimer [*µ*g/L]	1683 (982–3293)	2076 (1300–4512)	2039 (975–4670)	<500
sNGAL [*µ*g/L]	117.1 (70.8–208.5)	170.9 (100.8–237.1)	166.7 (102.2–216.6)	F: 21.6–276.0/M: 14.4–169.2
uNGAL [*µ*g/L]	28.5 (15.5–57.0)	38.3 (16.5–95.3)	41.7 (17.0–65.1)	<131.7
uACR [mg/g]	43.3 (21.0–79.4)	35.8 (20.8–89.5)	22.2 (14.2–92.3)	<30

Data are presented as median (lower–upper quartile); ^*∗*^reference values established in 21 healthy controls; Ang-2: angiopoietin-2; CRP: C-reactive protein; WBC: white blood cells; HCT: hematocrit; PLT: platelets; NGAL: neutrophil gelatinase-associated lipocalin in (s) serum and (u) urine; uACR: urine albumin/creatinine ratio.

**Table 3:** Angiopoietin-2 serum concentrations as a predictor of the severity of acute pancreatitis—results of simple logistic regression.

Dependent variable	OR (95% CI), per 1 ng/mL increase in Ang-2 concentration		
	24 h	48 h	72 h
SAP	1.11 (1.01–1.22)	1.22 (1.03–1.46)	1.31 (1.07–1.60)
SAP or MSAP	1.29 (1.03–1.62)	1.58 (1.18–2.10)	1.70 (1.21–2.38)
BISAP in the first 24 hours ≥3	1.18 (1.04–1.33)	1.37 (1.12–1.68)	1.68 (1.21–2.20)
SIRS	1.25 (1.08–1.44)	1.49 (1.17–1.90)	1.48 (1.17–1.88)
AKI (KDIGO)	1.12 (1.02–1.24)	1.37 (1.12–1.68)	1.49 (1.17–1.90)
Renal failure (MMSS)	1.10 (1.01–1.21)	1.28 (1.08–1.52)	1.44 (1.15–1.80)
Pleural effusion	NS	1.25 (1.06–1.47)	1.33 (1.10–1.63)
Death	NS	1.27 (1.02–1.59)	1.42 (1.07–1.89)

For abbreviations, see Table 1; AKI: acute kidney injury.

**Table 4:** Simple correlations between angiopoietin-2 and selected variables during the first 72 hours of acute pancreatitis.

Variable	24 h		48 h		72 h	
	*R*	*p*	*R*	*p*	*R*	*p*
Age	0.27	0.033	0.26	0.035	0.26	0.036
Urea	0.36	0.003	0.41	<0.001	0.34	0.004
Creatinine	0.30	0.014	0.34	0.006	0.08	NS
CRP	0.48	<0.001	0.31	0.010	0.35	0.004
Albumin	−0.23	NS	−0.45	<0.001	−0.48	<0.001
Calcium	−0.10	NS	−0.44	<0.001	−0.43	<0.001
HCT	−0.30	0.014	−0.36	0.003	−0.39	0.001
Fibrinogen	0.28	0.024	0.26	0.034	0.46	<0.001
D-dimer	0.40	0.001	0.44	<0.001	0.38	0.001
sNGAL	0.53	<0.001	0.52	<0.001	0.50	<0.001
uNGAL	0.59	<0.001	0.57	<0.001	0.52	<0.001
uACR	0.29	0.022	0.33	0.009	−0.01	NS

Ang-2: angiopoietin-2; HCT: hematocrit; NGAL: neutrophil gelatinase-associated lipocalin in (s) serum and (u) urine; CRP: C-reactive protein; uACR: urine albumin/creatinine ratio.

## References

[1] Mentula P., Leppäniemi A. (2014). Position paper: timely interventions in severe acute pancreatitis are crucial for survival. *World Journal of Emergency Surgery*.

[2] El-Banawy H. S., Gaber E. W., Maharem D. A., Matrawy K. A. (2012). Angiopoietin-2, endothelial dysfunction and renal involvement in patients with systemic lupus erythematosus. *Journal of Nephrology*.

[3] Tenner S., Baillie J., Vege S. S. (2013). American College of Gastroenterology guideline: management of acute pancreatitis. *The American Journal of Gastroenterology*.

[4] Petejova N., Martinek A. (2013). Acute kidney injury following acute pancreatitis: a review. *Biomedical Papers*.

[5] Buter A., Imrie C. W., Carter C. R., Evans S., McKay C. J. (2002). Dynamic nature of early organ dysfunction determines outcome in acute pancreatitis. *British Journal of Surgery*.

[6] Mentula P., Kylänpää-Bäck M.-L., Kemppainen E. (2003). Decreased HLA (human leucocyte antigen)-DR expression on peripheral blood monocytes predicts the development of organ failure in patients with acute pancreatitis. *Clinical Science*.

[7] Mole D. J., Olabi B., Robinson V., Garden O. J., Parks R. W. (2009). Incidence of individual organ dysfunction in fatal acute pancreatitis: analysis of 1024 death records. *HPB*.

[8] Zhou J., Li Y., Tang Y. (2015). Effect of acute kidney injury on mortality and hospital stay in patient with severe acute pancreatitis. *Nephrology*.

[9] Sporek M., Kolber W., Pedziwiatr M., Kuzniewski M., Walocha J., Kusnierz-Cabala B. (2015). Prediction of severe acute pancreatitis—selected prognostic scales and laboratory markers useful in the early stage of the disease. *Przegląd Lekarski*.

[10] Buddingh K. T., Koudstaal L. G., Van Santvoort H. C. (2014). Early angiopoietin-2 levels after onset predict the advent of severe pancreatitis, multiple organ failure, and infectious complications in patients with acute pancreatitis. *Journal of the American College of Surgeons*.

[11] Whitcomb D. C., Muddana V., Langmead C. J. (2010). Angiopoietin-2, a regulator of vascular permeability in inflammation, is associated with persistent organ failure in patients with acute pancreatitis from the United States and Germany. *The American Journal of Gastroenterology*.

[12] Watanabe T., Tsuji Y., Kodama Y., Isoda H., Yamamoto H., Chiba T. (2011). Relationship between serum angiopoietin-2 level and perfusion CT parameters in severe acute pancreatitis. *American Journal of Gastroenterology*.

[13] Thurston G., Daly C. (2012). The complex role of angiopoietin-2 in the angiopoietin—Tie signaling pathway. *Cold Spring Harbor Perspectives in Medicine*.

[14] Tsai Y.-C., Chiu Y.-W., Tsai J.-C. (2014). Association of angiopoietin-2 with renal outcome in chronic kidney disease. *PLoS ONE*.

[15] Banks P. A., Bollen T. L., Dervenis C., Gooszen H. G., Johnson C. D., Sarr M. G. (2013). Classification of acute pancreatitis—2012: revision of the Atlanta classification and definitions by international consensus. *Gut*.

[16] Kellum J. A., Lameire N., Aspelin P. (2013). Diagnosis, evaluation, and management of acute kidney injury: a KDIGO summary (Part 1). *Critical Care*.

[17] Petrov M. S., Shanbhag S., Chakraborty M., Phillips A. R. J., Windsor J. A. (2010). Organ failure and infection of pancreatic necrosis as determinants of mortality in patients with acute pancreatitis. *Gastroenterology*.

[18] (2013). KDIGO 2012 clinical practice guideline for the evaluation and management of chronic kidney disease. *Kidney International*.

[19] Kümpers P., Hafer C., David S. (2010). Angiopoietin-2 in patients requiring renal replacement therapy in the ICU: relation to acute kidney injury, multiple organ dysfunction syndrome and outcome. *Intensive Care Medicine*.

[20] Fiedler U., Krissl T., Koidl S. (2003). Angiopoietin-1 and angiopoietin-2 share the same binding domains in the Tie-2 receptor involving the first Ig-like loop and the epidermal growth factor-like repeats. *The Journal of Biological Chemistry*.

[21] Yamakawa M., Liu L. X., Date T. (2003). Hypoxia-inducible factor-1 mediates activation of cultured vascular endothelial cells by inducing multiple angiogenic factors. *Circulation Research*.

[22] Szederjesi J., Almasy E., Lazar A., Hutanu A., Georgescu A. (2015). The role of angiopoietine-2 in the diagnosis and prognosis of sepsis. *The Journal of Critical Care Medicine*.

[23] Luz Fiusa M. M., Costa-Lima C., de Souza G. R. (2013). A high angiopoietin-2/angiopoietin-1 ratio is associated with a high risk of septic shock in patients with febrile neutropenia. *Critical Care*.

[24] Soto K., Papoila A. L., Coelho S. (2013). Plasma NGAL for the diagnosis of AKI in patients admitted from the emergency department setting. *Clinical Journal of the American Society of Nephrology*.

[25] Zhao J. G., Liao Q., Zhao Y. P., Hu Y. (2014). Mortality indicators and risk factors for intraabdominal hypertension in severe acute pancreatitis. *International Surgery*.

[26] Neyra J. A., Li X., Yessayan L., Adams-Huet B., Yee J., Toto R. D. (2015). Dipstick albuminuria and acute kidney injury recovery in critically ill septic patients. *Nephrology*.

[27] Grams M. E., Sang Y., Ballew S. H. (2015). A meta-analysis of the association of estimated GFR, albuminuria, age, race, and sex with acute kidney injury. *American Journal of Kidney Diseases*.

[28] James M. T., Grams M. E., Woodward M. (2015). A meta-analysis of the association of estimated GFR, albuminuria, diabetes mellitus, and hypertension with acute kidney injury. *American Journal of Kidney Diseases*.

[29] Chang F.-C., Lai T.-S., Chiang C.-K. (2013). Angiopoietin-2 is associated with albuminuria and microalbuminuria in chronic kidney disease. *PLoS ONE*.

